# Phytochemical Analysis and Antifungal Activity of Extracts from Leaves and Fruit Residues of Brazilian Savanna Plants Aiming Its Use as Safe Fungicides

**DOI:** 10.1007/s13659-016-0101-y

**Published:** 2016-05-11

**Authors:** Caroline Alves Breda, Alessandra Marcon Gasperini, Vera Lucia Garcia, Karin Maia Monteiro, Giovana Anceski Bataglion, Marcos Nogueira Eberlin, Marta Cristina Teixeira Duarte

**Affiliations:** School of Food Engineering, State University of Campinas, UNICAMP, Monteiro Lobato Street, 80, Barão Geraldo, Campinas, São Paulo 13083-862 Brazil; Microbiology Division of Research Center for Chemistry, Biology and Agriculture – CPQBA, University of Campinas, UNICAMP, Alexandre Cazelatto Street, 999, Betel, Paulínia, São Paulo 13148-218 Brazil; Organic Chemistry and Pharmaceutical Division of Research Center for Chemistry, Biology and Agriculture – CPQBA, University of Campinas, UNICAMP, Alexandre Cazelatto Street, 999, Betel, Paulínia, São Paulo 13148-218 Brazil; Pharmacology and Toxicology Division of Research Center for Chemistry, Biology and Agriculture – CPQBA, University of Campinas, UNICAMP, Alexandre Cazelatto Street, 999, Betel, Paulínia, São Paulo 13148-218 Brazil; ThoMSon Mass Spectrometry Laboratory, Institute of Chemistry, State University of Campinas, UNICAMP, Campinas, São Paulo 13084-971 Brazil

**Keywords:** Brazilian savanna fruits, Residues, Natural fungicides, Phytopathogens

## Abstract

**Abstract:**

The increasing demand for safe food without preservatives or pesticides residues has encouraged several studies on natural products with antifungal activity and low toxicity. In this study, ethanolic extracts from leaves and fruit residues (peel and seeds) of three Brazilian savanna species (*Acrocomia aculeata*, *Campomanesia adamantium* and *Caryocar brasiliense*) were evaluated against phytopathogenic fungi. Additionally, the most active extract was chemically characterized by ESI-MS and its oral acute toxicity was evaluated. Extracts from *C. brasiliense* (pequi) peel and leaves were active against *Alternaria alternata*, *Alternaria solani* and *Venturia pirina* with minimal inhibitory concentrations between 350 and 1000 µg/mL. When incorporated in solid media, these extracts extended the *lag* phase of *A. alternata* and *A. solani* and reduced the growth rate of *A. solani*. Pequi peel extract showed better antifungal activity and their ESI-MS analysis revealed the presence of substances widely reported as antifungal such as gallic acid, quinic acid, ellagic acid, glucogalin and corilagin. The oral acute toxicity was relatively low, being considered safe for use as a potential natural fungicide.

**Graphical Abstract:**

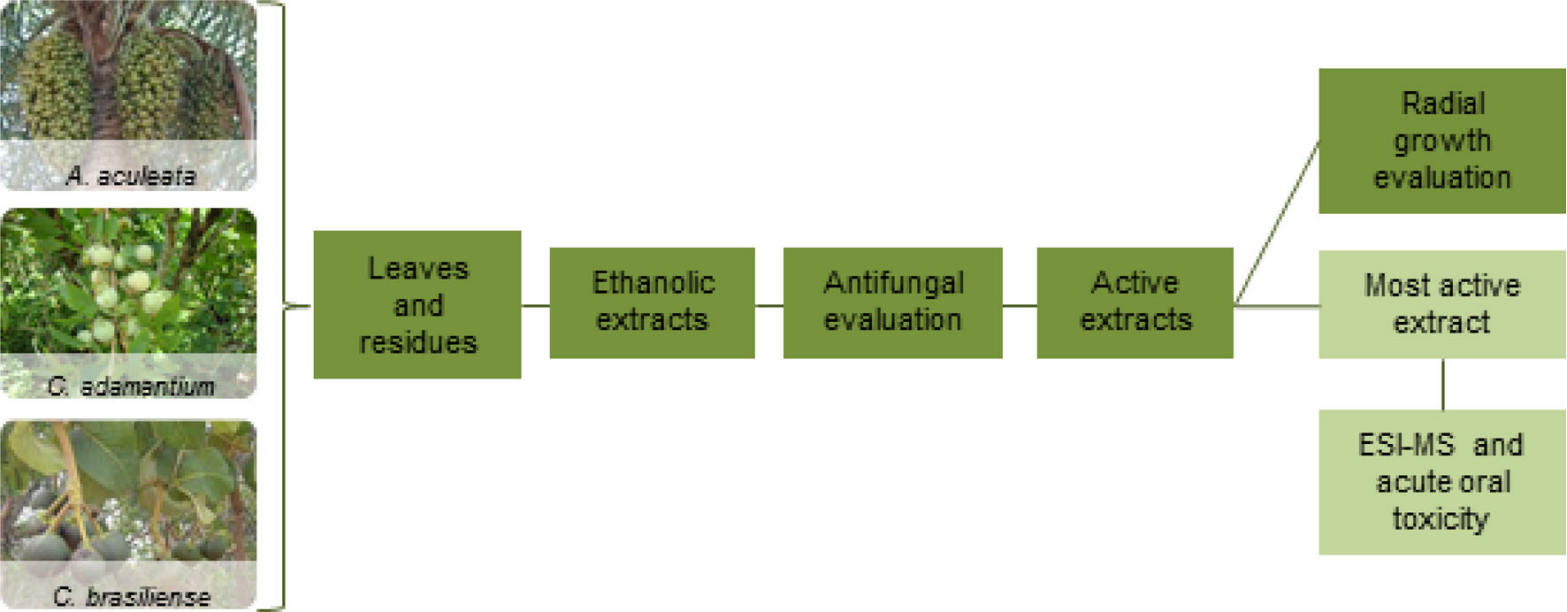

## Introduction

The most important phytopathogens include bacteria, fungi, nematodes and viruses, which can attack different crops resulting in considerable economic losses. Among them, fungi represent the largest group of these pathogens [1] and can contaminate fruits and vegetables from cultivation to harvest, during transportation, storage and processing [2]. Synthetic preservatives and fungicides have been used for decades to control fungal spoilage, however, the indiscriminate use of these substances has caused health problems for humans and animals due their carcinogenicity, teratogenicity and acute toxicity [3], environmental contamination, besides causing resistance in pathogens [4]. Thus, investigators are seeking safer alternatives to replace synthetic compounds used as fungicides and preservatives. Environmentally friendly antifungal agents, such as plant extracts have shown great potential to replace synthetic products due their low cost, local availability, lack of toxicity and biodegradability [5].

Brazil is one of the countries that present an abundant plant diversity distributed in several biomes [6]. The Brazilian savanna is the second largest biome in South America and its flora can be considered as a renewable natural resource. The proper management of this environment can result in permanent human occupation providing raw material to the industry and helping in the biodiversity preservation [7]. The fruit species inserted in the Brazilian savanna flora have great potential for use in agriculture [8] due to their high nutritional value, peculiarity of sensory attributes such as flavour and intense aroma [9]. Among the important species are *Acrocomia aculeata* (macaúba), *Campomanesia adamantium* (guavira) and *Caryocar brasiliense* (pequi) that are already widely used in food and folk medicine. Some studies showed the antimicrobial potential of fruits and leaves of *C. brasiliense* against *Cryptococcus neoformans* [10] and leaves of *C. adamantium* against *Escherichia coli*, *Candida albicans*, *S. aureus* and *Pseudomonas aeruginosa* [11]. However, few studies addressed the potential for using of the peel and seed of these fruits that are usually discarded after processing.

Thus, in this study we assessed in vitro antifungal activity against phytopathogenic fungi using plant extracts obtained from leaves and fruit residues of some Brazilian savanna species. In addition, the extract with the best activity was submitted to chemical characterization by ESI-MS and acute oral toxicity evaluation.

## Results and Discussion

### Extracts Yields

Yields of crude extracts (%) obtained from leaves and residues of Brazilian savanna fruits are shown in Table 1. The yields of the extracts obtained in this study varied according to sample used in extraction as peels, seeds and leaves. The highest yield was observed for the crude extract of guavira seeds (64.16 %) followed by crude extracts obtained from pequi leaves (41.97 %) and pequi peels (26.29 %). The lowest yield was observed for the crude extract of macaúba leaves (7.07 %). According to Dapkevicius et al. the amount of material extracted can be influenced by chemical composition of substrate and extraction technique [12].

**Table 1:** Yields of ethanolic extracts obtained from leaves and residues of Brazilian savanna fruits

Plant specie (common name)	Used parts	Yield (%, dry mass)
Macaúba	Leaves	7.07
	Peels	21.03
Pequi	Leaves	41.97
	Peels	26.29
Guavira	Leaves	18.69
	Peels	16.96
	Seeds	64.16

### Antifungal Activity of the Extracts

Antifungal activity of the crude extracts evaluated in the concentration range from 2000 to 1.95 µg/mL was only observed for the extracts obtained from pequi (peels and leaves) against the fungi *A. alternata*, *A. solani* and *V. pirina.* Extracts obtained from pequi peels showed MIC of 350 µg/mL for *A. solani* and *V. pirina* and 500 µg/mL for *A. alternata*, while extracts from pequi leaves showed higher MIC values, namely 1000 µg/mL for *A. alternata* and *A. solani* and 400 µg/mL for *V. pirina.* Thus, according to proposed by Duarte et al. [13] the extract from pequi peels and leaves can be considered as strongly active against *V. pirina*, and moderately active for *A. alternata* and *A. solani*. This behavior probably is attributed by the higher concentration of phytochemicals such as phenolic compounds in pequi peels than leaves. Bashir and Abu-Goukh [14] found that the fruits peels are rich in phenolic compounds, which probably can be assigned to the protective function of the external factors that they exert on fruit, as a way to avoid insects attacks and microbial diseases, ensuring the propagation of the species.

Regarding to MFC, only the extract of pequi peels showed activity against *A. solani*, at concentration of 1000 µg/mL, whose MFC:MIC ratio was 2.86:1.0, which according to Dolan and Costerton [15] defines the action of the extract as fungistatic.

### Radial Growth of Fungi

**Fig. 1:**
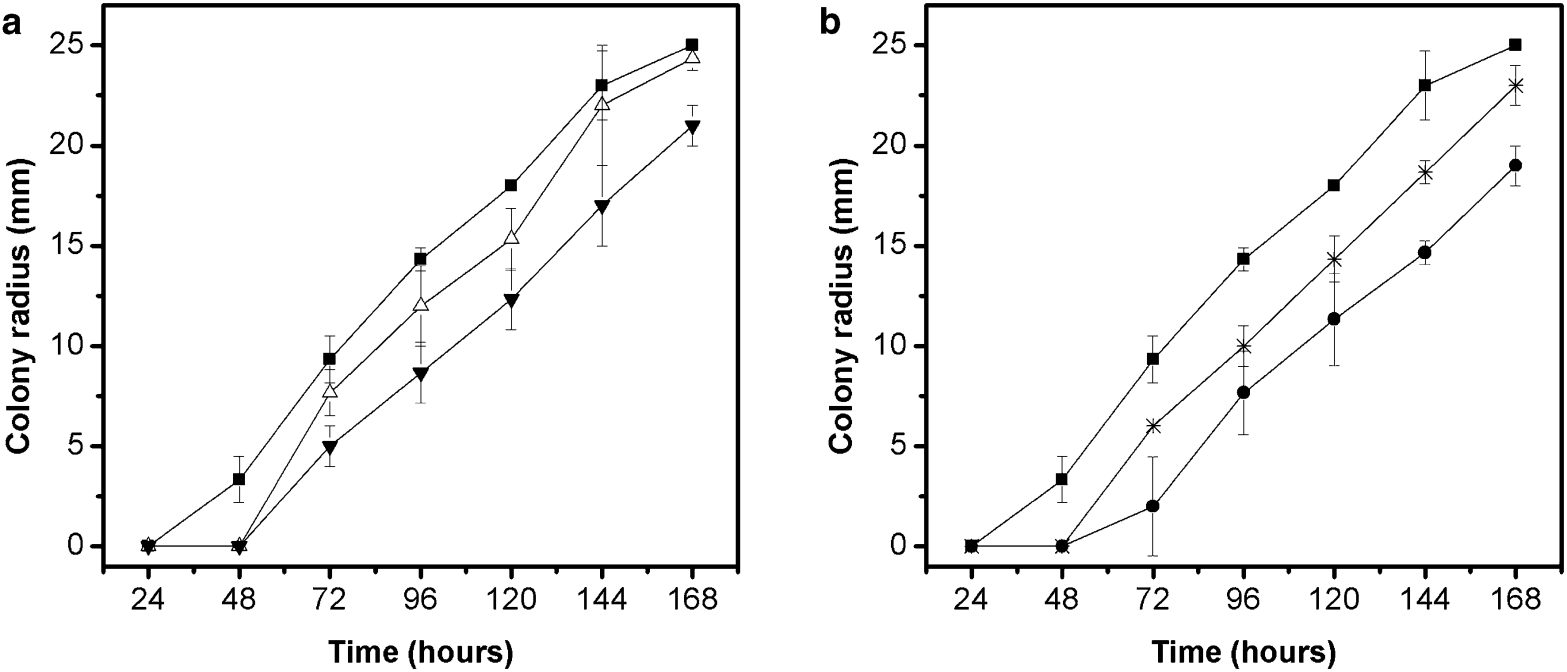
Radial growth of the fungus *A. alternata* in MEA medium containing different concentrations of extracts from pequi peels (**a**) and pequi leaves (**b**). Control: medium without extract (); Medium containing extract from pequi peels at concentrations of 500 () and 1000 () µg mL^−1^; Medium containing extract from pequi leaves at concentrations of 1000 () and 2000 () µg mL^−1^

Based on pre-determined criteria, extracts from pequi leaves (PL) and peels (PP) were chosen to evaluate the effects on radial growth of *A. alternata*, *A. solani* and *V. pirina*. Average values of colony radius versus growth time of *A. alternata* on malt extract agar (MEA) incorporated with extracts from pequi peels (500 and 1000 µg/mL) and leaves (1000 and 2000 µg/mL) are shown in Figs. 1a, b. The growth curves show an initial *lag* phase (adaptation) that had a longer time in the presence of the extracts (48 h) than in the control assay (24 h), followed by a linear growth. Moreover, it can be observed that at the end of experiment, the assays with higher concentrations of extracts (PP 1000 and PL 2000 µg/mL) showed lower colony radius (21.00 and 19.00 mm, respectively) when compared with the control assay (25.00 mm) (p < 0.05).

Applying the linear model in radial growth curves were obtained the predicted values of *lag* phase (λ) and radial growth rate (µ) for *A. alternata* in the assays containing pequi extracts and without extract (control). The coefficients of determination (R^2^) obtained were between 0.97 and 0.98, which according to Baert et al. [16] indicates a good fit. The average values and their standard deviations for each parameter are shown in Table 2a. As it can be seen, the duration of the *lag* phase (λ) predicted by the model was significantly affected (p < 0.05) in the PP 1000, PL 1000 and PL 2000 µg/mL assays when compared to control assay, while the PP 500 µg/mL assay showed no statistical differences. However, the comparison of the assays containing extracts showed no statistically significant differences, indicating that both high and low extracts concentrations present the same effect in delaying the start of the linear growth.

**Table 2:** Predicted values by linear fit for *lag* phase duration (λ) and radial growth rate (µ) of *A. alternata* (a), *A. solani* (b), and *V. pirina* (c) in culture medium containing different concentrations of extracts from pequi peel (PP) and pequi leaves (PL)

Assay	λ (h)	µ (mm/h)
(a) *A. alternata*		
PP 500 µg/mL	40.30 ± 3.02^ab^	0.1906 ± 0.002^a^
PP 1000 µg/mL	46.10 ± 4.49^b^	0.1715 ± 0.012^a^
PL 1000 µg/mL	43.95 ± 0.51^b^	0.1854 ± 0.007^a^
PL 2000 µg/mL	57.61 ± 16.62^b^	0.1739 ± 0.018^a^
Control	20.93 ± 3.97^a^	0.1709 ± 0.005^a^
(b) *A. solani*		
PP 350 µg/mL	41.81 ± 1.99^b^	0.1585 ± 0.002^b^
PP 700 µg/mL	54.58 ± 2.69^b^	0.1617 ± 0.005^b^
PP 1000 µg/mL	91.99 ± 8.79^d^	0.1849 ± 0.012^c^
PL 1000 µg/mL	72.29 ± 2.20^c^	0.1566 ± 0.008^b^
PL 2000 µg/mL	75.78 ± 3.31^c^	0.1085 ± 0.011^a^
Control	22.78 ± 6.14^a^	0.1493 ± 0.005^b^
(c) *V. pirina*		
PP 350 µg/mL	11.11 ± 1.51^a^	0.0892 ± 0.001^a^
PP 700 µg/mL	14.58 ± 4.18^a^	0.0966 ± 0.011^a^
PL 400 µg/mL	12.24 ± 1.88^a^	0.0941 ± 0.003^a^
PL 800 µg/mL	16.95 ± 4.29^a^	0.0942 ± 0.001^a^
Control	13.23 ± 0.50^a^	0.0947 ± 0.013^a^

Different letters in the same column (for each fungus) indicate significant difference according to Tukey’s test at the 5 % level

In the radial growth rate (µ), there was no statistically significant difference between assays containing extracts and without extracts (p > 0.05), indicating that major effect of the extracts addition occurred in adaptation phase of the fungus. This behaviour may be attributed to the presence of phenolic compounds in the evaluated extracts that can act denaturing spore germination related enzymes, or interfering with amino acids associated to germination process [17].

**Fig. 2:**
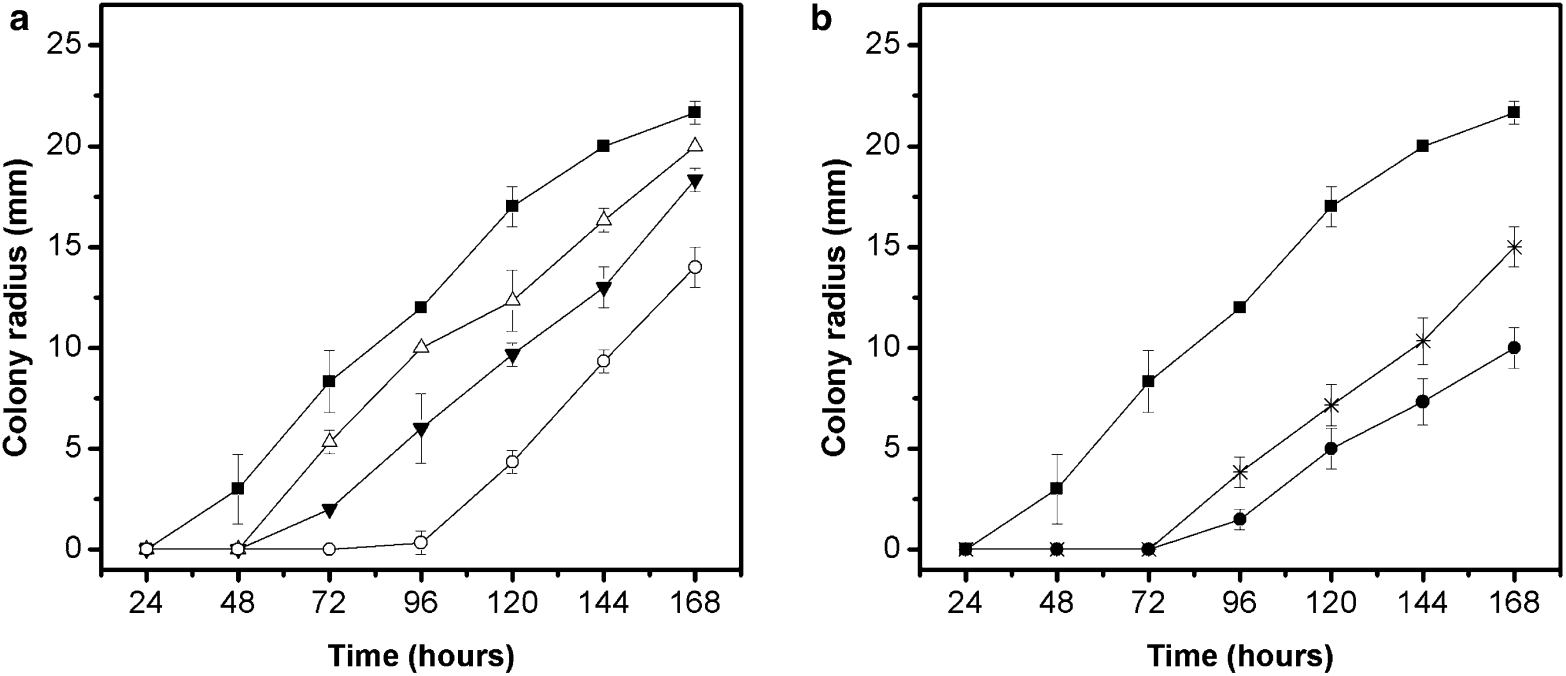
Radial growth of the fungus *A. solani* in MEA medium containing different concentrations of extracts from pequi peels (**a**) and pequi leaves (**b**). Control: medium without extract (); Medium containing extract from pequi peels at concentrations of 350 (), 700 () and 1000 () µg mL^−1^; Medium containing extract from pequi leaves at concentrations of 1000 () and 2000 () µg mL^−1^

The radial growth curves of *A. solani* in MEA plus extracts from pequi peels (concentrations of 350,700 and 1000 µg/mL) and leaves (concentrations of 1000 and 2000 µg/mL) are shown in Figs. 2a, b. The *A. solani* growth in presence of extracts showed a similar profile to *A. alternata* due to presence of the initial adjustment phase with a subsequent linear growth. The duration of this adaptation period varied according to extracts concentration being larger in the assays PP 1000, PL 1000 and PL 2000 µg/mL, with early fungal growth after 72 h. The maximum growth was reached at 168 h for PP 700, PP 1000, PL 1000 and PL 2000 µg/mL assays. The colony radius in these assays were 18.33, 14.00, 15.00 and 10.00 mm, respectively, showing lower values (p < 0.05) compared with the control assay (21.67 mm). Extract from pequi leaves at 2000 µg/mL was therefore more effective in reducing the colony radius than other conditions tested.

Linear fit of radial growth curves of *A. solani* showed coefficients of determination between 0.98 and 0.99, characterizing this model as appropriate to predict λ and μ values for the different assays (Table 2b). The results show that all tests with pequi extracts incorporated into the culture medium had longer *lag* phase when compared with control assay. Moreover, the fungal growth was influenced by both extract used and its concentration. This fact is evidenced by the statistical analysis, since extracts from pequi peels and leaves used at same concentrations (PP 1000 and PF 1000 µg/mL) showed significative differences (p < 0.05). Another evidence is the smallest λ value for the assay with the highest concentration of pequi leaves extract (PL 2000 µg/mL), also in comparison with the assay whose *lag* phase duration was longer.

Regarding the radial growth rate (µ), there was no statistical difference (p > 0.05) between the assays PP 350, PP 700, PL 1000 µg/mL and control. The lowest growth rate was detected in the assay PL 2000 µg/mL, whereas the highest growth rate (0.1849 mm/h) was detected in the assay using the highest concentration of pequi peels extract (PP 1000 µg/mL). These results support the hypothesis that the extracts may act effectively only increasing the duration of *lag* phase.

**Fig. 3:**
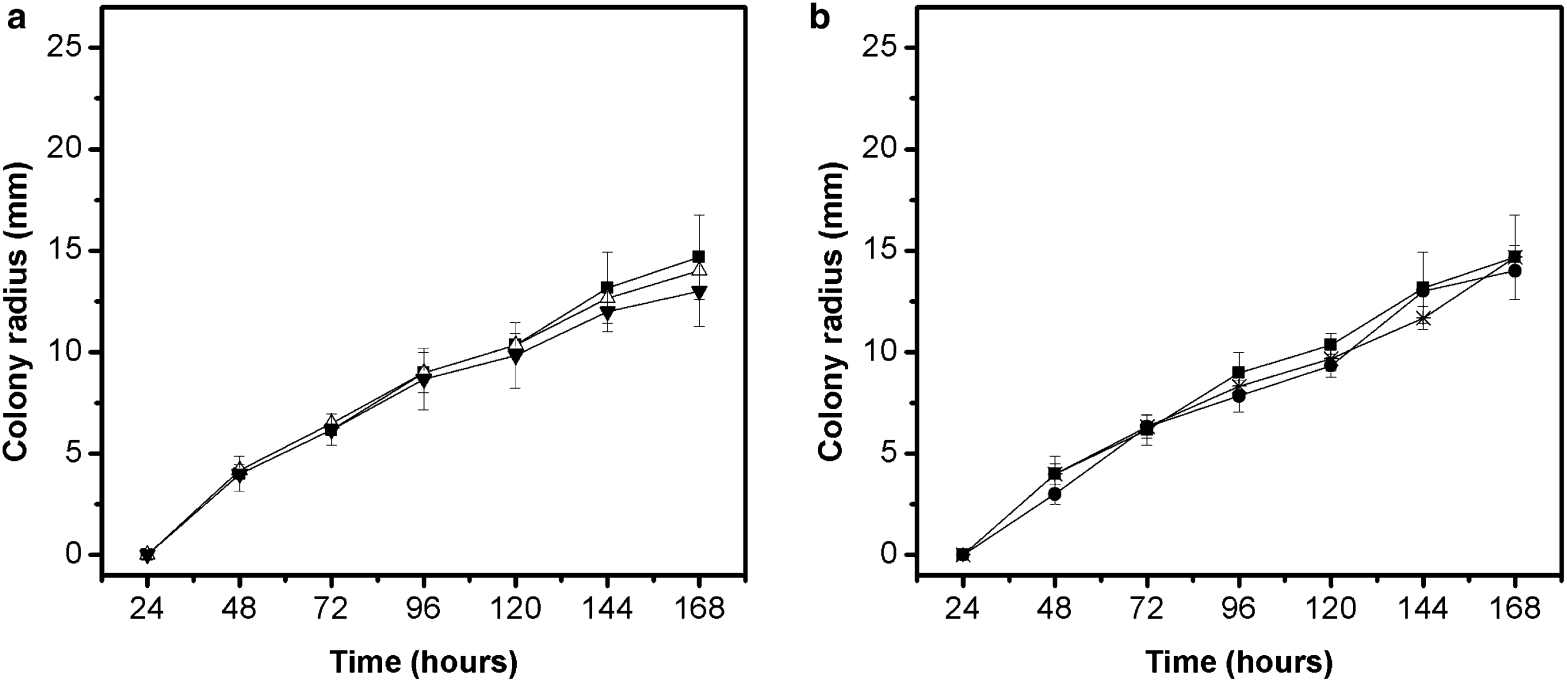
Radial growth of the fungus *V. pirina* in PDA medium containing different concentrations of extracts from pequi peels (**a**) and pequi leaves (**b**). Control: medium without extract (); Medium containing extract from pequi peels at concentrations of 350 () and 700 () µg mL^−1^; Medium containing extract from pequi leaves at concentrations of 400 () and 800 () µg mL^−1^

Radial growth curves of *V. pirina* in potato dextrose agar (PDA) without extracts (control assay) and plus the extracts are shown in Figs. 3a, b. Different from the behaviour observed in radial growth of *A. alternata* and *A. solani*, the addition of extracts did not delay the beginning of linear growth when compared with the control assay, which means less time for adaptation of these microorganisms to the medium. There was no statistically differences between all assays (p > 0.05) from the beginning of growth up to the maximum radius growth of the colonies at 168 h (7 days).

The adjustments made by the linear model provided R^2^ values between 0.97 and 0.98. The predicted values of λ and µ, as well their standard deviations in assays performed with *V. pirina* are shown in Table 2c. Analysing the results, there was no significant difference between the predicted values of *lag* phase duration and radial growth rate in the different assays (with and without extracts).

### Chemical Analysis

Due to its best antifungal activity, pequi peel extract was chemically characterized by ESI-MS in the negative ion mode, revealing the presence of 10 constituents, which were tentatively identified based on exact values of *m/z* and comparisons with fragmentation profiles. For gallic, quinic and ellagic acids, the fragmentation profiles were compared with those obtained for authentic standards, and for the other compounds with those previously described in literature [18–21]. Detailed identification is described in Table 3.

**Table 3:** Compounds tentatively identified in pequi peel extract using ESI–MS in the negative ion mode

[M–H]^−^	Tentative identification	Measured *m/z*	Calculated *m/z*	Error (ppm)	ESI–MS/MS
C_7_H_5_O_5_	Gallic acid	169.01420	169.01425	−0.30	79, 125
C_7_H_11_O_6_	Quinic acid	191.05600	191.05611	−0.57	85, 93, 127
	Brevifolin carboxylic acid fragment	247.02462			191, 219
C_13_H_7_O_8_	Brevifolin carboxylic acid	291.01452	291.01464	−0.41	247, 219, 191
C_14_H_5_O_8_	Ellagic acid	300.99919	300.99899	0.66	145, 284
C_13_H_15_O_10_	Glucogallin	331.06673	331.06707	−1.03	169, 241
C_19_H_13_O_12_	Ellagic acid xyloside	433.04144	433.04125	0.44	301
C_20_H_15_O_12_	Ellagic acid rhamnoside	447.05902	447.05690	4.74	301
C_20_H_15_O_13_	Ellagic acid glucoside	463.05293	463.05181	2.42	301
C_27_H_21_O_18_	Corilagin	633.07152	633.07334	−2.87	301
C_41_H_27_O_27_	Geraniin	951.06917	951.07452	−5.63	301

Among the identified compounds, only gallic and quinic acids were previously described in pequi peel extracts as phenolic compounds quercetin and quercetin-O-arabinose [21]. The variation of chemical composition of the extracts can be explained by environmental and genetic differences between plant materials used and the analysis methods employed.

Gallic acid (*m/z* 169.01420) is one of the simplest polyphenols found in plant tissues in its free form and as other compounds, such as tannins, ellagitannins, epigallocatechin-3-gallate, among others [22]. According to Bhouri et al. besides acting in free radicals scavenging, gallic acid has anti-inflammatory and anticancer properties and may also be used as an antioxidant for foods, cosmetics and pharmaceuticals [23]. Regarding the antifungal activity, Osorio et al. found that gallic acid at concentration of 1000 mg/L showed fungicidal effect against *Colletotrichum truncatum* and fungistatic against *A. alternata*, *Fusarium sambucinum* and *F. Verticilioides* [24].

Gallic acid and quinic acid (*m/z* 191.05600) are found in plants both in free form and in various esters [25]. Chen et al. reported antifungal activity for quinic acid isolated from a buthyl alcohol fraction of the ethanolic extract of *Araucaria cunninghamii* at 100 µg/mL that was able to inhibit the growth of *Helminthosporium sativum* in 9.3 % [26].

Brevifolin carboxylic acid (*m/z* 291.01452) is a compound widely used in tradicional Chinese medicines. Among their reported properties, are the antiviral activity against hepatitis B and growth and replication inhibition of tumors [27]. A study made by N’Guessan et al. showed a weak activity of brevifolin carboxylic acid against *Salmonella enteritidis*, *S. abony* and *S. typhimurium* [28].

Ellagic acid is a polyphenol which can occur in free form, glycoside or linked as ellagitannins esterified with glucose [29]. In this study, were identified in pequi peel extract free ellagic acid (*m/z* 300.99919) as well as ellagic acid glycoside in forms of ellagic acid xyloside (*m/z* 433.04144), rhamnoside (*m/z* 447.05902) and glucoside (*m/z* 463.08493). According to Osorio et al. ellagic acid at 1000 mg/L presented fungicidal activity against the phytopathogens *Phytium* sp. and *Colletotrichum coccodes*, and fungistatic against *C. truncatum*, *Fusarium solani*, *F. sambucinum* and *Rhizoctonia solani* [24].

Glucogallin (*m/z* 331.06673) was reported as a key metabolite in the synthesis of hydrolysable tannins, which are widely distributed in vegetal kingdom [30]. Latté and Kolodziej observed that glucogallin isolated from *Pelargonium reniform* showed activity also against *Microsporum gypseum*, *C. glabrata*, *C. krusei* and *C. neoformans*, however, no data were found in literature concerning the activity against plant pathogens [31].

Another compound identified in ethanolic extract of pequi peel was corilagin (*m/z* 633.07152), which presents antifungal activity reported in literature. According to Wang et al. this tannin had a broad pharmacological spectrum acting as antihypertensive, antiatherogenic, antitumoral, and antithrombotic, and it presents synergistic effect with β-lactams against methicillin-resistant *S. aureus* [32]. In relation to its effect as an antifungal, corilagin showed activity against *M. gypseum*, *C. glabrata*, *C. krusei*, *C. albicans* and *C. neoformans* [31].

The elagitannin geraniin (*m/z* 951.06917) is a crystalline substance with low astringency and properties that include free radicals scavenging acting as hepatoprotective, antiviral antihypertensive, anti-ulcer [33]. Taguri et al. found that geraniin isolated from leaves of *Elaeocarpus sylvestris* showed activity against different strains of *Salmonella*, *E. coli*, *S. aureus* and *Vibrio*, with MIC values ranged between 25 and 3200 µg/mL [34].

### Acute Oral Toxicity

The acute administration of pequi peel extract at 5000 mg/Kg produced lethality in three out of five animals within the first 48 h after dosing. As the animals were found dead it was not possible to perform gross necropsy. The two remaining moribund animals were removed from the study and humanely killed as they displayed severe distress. The macroscopic evaluation of their vital organs revealed no toxicity-related alterations.

However, the lower dose of 2000 mg/Kg produced no mortality or clinical signs of toxicity during the entire experimental period and no changes in body weight gain were observed comparing with both “naïve” and negative control groups. The gross evaluation of the vital organs showed no significant macroscopic changes. Finally, there was no statistical differences (p < 0.05) in the relative organ weight among the groups (data not shown).

Thus, pequi peel extract presented relatively low acute toxicity hazard and could be classified in the category defined by 2000 mg/Kg < LD_50_ < 5000 mg/Kg (Category 5 in the GHS) [35], being considered safe for use as food preservative or fungicide to control phytopatogenic fungi.

## Experimental Section

### General Experimental Procedures

Fungal spore suspensions were standardized on a Shimadzu UV Mini 1240 spectrophotometer (Kyoto, Japan). ESI-MS was performed on an Agilent 6550 iFunnel Q-TOF LC/MS system (Santa Clara, USA) constituting of an electrospray ionization source and a hybrid mass analyser (quadrupole-time of flight). Ethanol 99.9 %, polivinilpirrolidone (PVP), Tween 20 and sodium chloride were purchased from Vetec Chemicals (Rio de Janeiro, Brazil). Methanol HPLC grade and standard compounds (gallic acid, quinic acid and ellagic acid) were purchased from Sigma-Aldrich (St. Louis, USA).

MEA was prepared using the following reagents acquired from Oxoid (Basingstoke, England): malt extract (20 g/L), peptone (5 g/L) and agar (15 g/L). Pea medium was prepared using frozen green peas (150 g/L) purchased at a local market, sucrose (20 g/L) purchased from Vetec Chemicals (Rio de Janeiro, Brazil) and agar (15 g/L) purchased from Oxoid (Basingstoke, England). Oat medium was prepared using oat flakes (100 g/L) purchased at a local market and agar (15 g/L) purchased from Oxoid (Basingstoke, England). PDA and RPMI-1640 broth were acquired from Oxoid (Basingstoke, England) and Cultilab (Campinas, Brazil), respectively.

### Plant Material

Fruits and leaves of *A. aculeata* (Arecaceae), *C. adamantium* (Myrtaceae) and *C. brasiliense* (Caryocaraceae) were collected in Itahum district of Dourados, Mato Grosso do Sul, Brazil (Latitude −22.076340, Longitude −55.142760), between November 2011 and January 2012. Voucher specimens were deposited at the herbarium of the Faculty of Biological Sciences of Federal University of Grande Dourados (UFGD). The identification numbers of the voucher specimens *A.aculeata*, *C. adamantium* and *C. brasiliense* were DDMS 2169, DDMS 4602 and DDMS 505, respectively.

All material was collected in the morning period, packed in plastic bags and immediately submitted to processing.

The authorization for access to genetic resources was granted by CNPq - National Council for Scientific and Technological Development—number 010361/2012-0.

### Processing of Plant Material

Plant material (fruits and leaves) was selected considering the degree of ripeness of the fruits (favorable to consumption) and the physical integrity (absence of injuries or damage caused by microbial spoilage), and washed with distilled water. After that, fruits were peeled and pulped, and the peels from all the fruits and seeds of guavira were fractionated to smaller sizes. Residues and leaves were dried at 40 °C for 24 h, grounded to a fine powder using a domestic blender and stored in low density polyethylene bags at 25 °C until use.

### Plant Extracts Preparation

Plant extracts were obtained from mixing 10 g of each plant powder with 500 mL of absolute ethanol, followed by agitation at 200 rpm in shaker at 25 °C during 3 h. Then, the extracts were filtered and the residues of the extraction were added to 500 mL of absolute ethanol and extracted as previously. Finally, the final residues were washed with 250 mL of ethanol and the filtered extracts were combined. The ethanol was removed from the extract by means of vacuum distillation at 30 °C using a rotary evaporator, resulting in crude extracts.

The yield of the crude extracts was calculated according to Eq. 1.

1 $${\text{R}}\,\% = \frac{{M_{Ext} \times 100\,\% }}{{D_{m} }}$$

where R %, represents the percentage of yield of the crude extract; M_ext_, mass obtained from crude extract (g) and D_m_, dry mass of the plant material used for the extract production (g), determined by drying of plant material at 70 °C for 24 h.

### Microorganisms and Maintenance

The plants extracts were evaluated against the following fungal strains: *Alternaria solani* CCT 2673, A*lternaria alternata* CCT 1250, *Botrytis cinerea* CCT 1252, *Colletotrichum gloeosporioides* CBMAI 0864, *Mucor hiemalis* CBMAI 0547, *Phytophthora infestans* (isolated from potato by Iharabras S A Chemical Industries) and *Venturia pirina* CCT 3166. The fungi strains were obtained at Tropical Cultures Collection (CCT) from Tropical Research Foundation “André Tosello”—Campinas, São Paulo, Brazil and from Brazilian Collection of Environmental and Industrial Microorganisms (CBMAI) at CPQBA/UNICAMP—Campinas, São Paulo, Brazil.

The strains of *A. solani* and *A. alternata* were maintained and grown in MEA; *V. pirina,C. gloeosporiodes* and *M. hiemalis* in PDA; *P. infestans* in pea medium and *B. cinerea* in oat medium. After inoculation, the cultures were incubated at 25 °C until sporulation (between 7 and 10 days), except *B. cinerea* that was maintained under photoperiod during 10 days at 25 °C.

### Determination of Antifungal Activity

#### Dilution of Plant Extracts in Polivinilpirrolidone (PVP)

An amount of 80 mg of crude extracts were dissolved in 0.016 g/mL ethanolic solution of PVP at proportion of 1:4 (crude extract:PVP, w/w), according to described by El-Arini and Leuenberger [36] with some slight modifications. After dissolution, the extracts in PVP were dried by vacuum distillation at 30 °C until total ethanol volatilization, resulting in crude extracts with PVP. An aliquot of 10 mL of RPMI-1640 broth was added in each flask containing crude extracts with PVP in order to obtain solutions at 8 mg/mL. All dissolved extracts were placed into glass flasks hermetically closed and stored at −22 °C until use.

#### Standardized Inoculum Preparation

The fungal inoculum was standardized according to NCCLS [37]. Petri dishes containing the sporulating fungal cultures were covered with 1 mL of sterilized solution of Tween 20 at 0.5 % (v/v) and gently homogenized with Drigalski spatel. The supernatant (spores suspension) was removed with a sterilized Pasteur pipette, filtered in cheesecloth to retain hyphae fragments and transferred to a sterilized glass tube.

An aliquot of spore suspension was analysed in spectrophotometer at 530 nm and adjusted with 0.85 % sodium chloride solution for an optical density between 0.09 and 0.11 (transmittance of 80–82 %). Finally, the standardized suspension was diluted in a proportion 1:50 (v/v) in RPMI-1640 broth, corresponding to approximately 0.4 × 10^4^ CFU/mL.

#### Minimal Inhibitory Concentration (MIC)

MIC test was performed using sterile 96-well microplates containing 100 µL/well of RPMI-1640 broth. In the first column were added 50 µL of diluted extract (samples sterility control). In the second column were placed 100 µL of each extract and serial dilutions were performed to obtain concentrations ranging from 2000 to 1.95 µg/mL. Thiofanate-methyl and RPMI-1640 broth were used as positive and negative controls, respectively. The standardized inoculum was added to all wells, except for the first column, the plates were incubated at 25 °C for 5 days. The MIC was defined as the lowest concentration of extract that inhibited microorganism visible growth [37].

#### Minimal Fungicidal Concentration (MFC)

In order to determine the MFC, an aliquot of 10 µL of each incubated well of MIC and the higher concentrations were sub-cultured on Petri dishes containing specific solid medium for each fungus and incubated at 25 °C for 5 days. The MFC was defined as the lowest concentration of extract that allowed no visible growth on the specific solid medium.

To determine the nature of the antifungal effect of extracts, the MFC:MIC ratio was calculated [15]. The extract was considered fungicidal when MFC:MIC ratio was between 1:1 and 2:1, and fungistatic when the ratio was higher than 2:1.

#### Evaluation of Radial Growth

Extracts that presented antifungal activity were diluted in PVP solution and sterile distilled water at 20 mg/mL (w/v) (stock solution). Aliquots of stock solution were added in flasks containing 25 mL of the adequate solid medium, previously sterilized and cooled between 45 °C and 50 °C, in order to obtain final concentrations according to MFC, MIC, and twice MIC.

Medium containing the extracts were homogenized and placed (6 mL) into 49 mm diameter Petri dishes. As control assays were prepared Petri dishes containing only the culture medium. After solidification, the medium of each plate was perforated to obtain a 2 mm diameter central orifice.

In each orifice of the plates were added 4 µL of standardized spore solutions of the respective fungi (prepared as item 2.5.2, with RPMI-1640 broth replaced by sterile distilled water). Then, the plates were incubated at 25 °C and the mycelial growth was evaluated every 24 h during 7 days by measurement of the fungal colony along two perpendicular radius (mm). Radial growth rate (mm.h^−1^) was calculated using linear predictive model [38] according to Eq. 2:

2 $$r = \mu (t - {{\lambda }})$$

where, *r* is the average radius of colony (mm), µ is the radial growth rate (mm/h), *t* is the time (h) and λ is the duration of the *lag* phase (h). This last parameter was obtained by calculation of corresponding time to the intersection point between the line obtained by model and the line parallel to the X-axis.

### Chemical Characterization of the Most Active Extract

Electrospray Ionization Mass Spectrometry (ESI-MS) was used to identify the compounds present in the pequi peel extract (the most active among the tested extracts). An amount of 1 mg of the pequi peel extract sample was diluted in 1 mL of methanol and the mass spectra were acquired in the negative ion mode in the *m/z* range of 100–1000. The extract solution was analysed by direct infusion with flow rate of 10 µL/min. The source ionization conditions were: gas temp: 280 °C, drying gas: 11 L/min, nebulizer: 30 psi, sheath gas temp: 300 °C, sheath gas flow: 12 L/min, Vcap: 3000 V, nozzle: 0 V, fragmentor: 150 V. For MS/MS analysis, the ions of interest were selected in the quadrupole analyser and transferred to the collision cell, where the collision energy was adjusted from 10 to 40 V, depending on the extension of fragmentation of the ion of interest. Then, the ion products were analysed by TOF analyser in the *m/z* range of 50 until a value slightly above that of the precursor ion. The software Agilent MassHunter (Agilent, Santa Clara, USA) was used to acquire and process the data.

### Acute Oral Toxicicity Evaluation of the Most Active Extract

In order to determine the safety of the most active extract, acute oral toxicity evaluation was carried out. Female mice (*Mus musculus,* Albinus, Swiss) at the age of 8 weeks, weighing 20–25 g and specific pathogen free were acquired from the Animal Experimental Centre of Campinas State University. The animals were kept in five animal groups in polyethylene boxes in a controlled environment (23 ± 2 °C, 53 ± 2 % RH), under a 12 h light/dark cycle with food and water ad libitum.

Acute oral toxicity evaluation was conducted according to OECD guideline number 420 by fixed dose method [35]. The animal testing was approved by the Committee of Ethics in Animal Experimentation (CEEA) of the University of Campinas—UNICAMP (record no 3360-1). For this study, the extract that showed better antifungal activity was solubilized in phosphate-saline buffer (PBS) and administered to single animals in a sequential manner in a total of 5 animals/dose level by gavage method. The initial dose level of 5000 mg/Kg was chosen according to the Criteria for Hazard Category 5 in the GHS (Global Harmonization System) [35]. Two additional groups with five animals each were used as “naïve” and negative control, respectively. The group of negative control was submitted to PBS (maximum volume 10 mL/Kg).

After dosing, each animal was closely observed for possible clinical signs during the first 30 min and then, periodically during the first 24 h. All animals were observed once daily thereafter for 14 days. Body weight was recorded weekly (days 7 and 14). At the end of the experiment (day 14), the surviving animals were weighed, euthanized by cervical dislocation and submitted to gross necropsy. Adrenal glands, spleen, liver and kidneys were extracted and weighed after macroscopic evaluation. The analysis of organ weights was performed considering the relative weight (body weight/organ weight relation).

### Statistical Analysis

The experiments were performed twice and in triplicate, except for chemical analysis and oral acute toxicity evaluation of the extract. The data obtained for radial growth evaluation and acute oral toxicity were statistically evaluated using variance analysis (ANOVA) and the means compared by Tukey’s test (p < 0.05) using version 8.0 of Statistica software (StatSoft, Tulsa, USA).

## References

[1] Camele I, Altieri L, de Martino L, de Feo V, Mancini E, Rana GL (2012). Int. J. Mol. Sci..

[2] Braghini R, Pozzi CR, Aquino S, Rocha LO, Corrêa B (2009). Appl. Radiat. Isot..

[3] Tian J, Ban X, Zeng H, Huang B, He J, Wang Y (2011). Food Control.

[4] de Rodríguez DJ, García RR, Castillo FDH, González CNA, Galindo AS, Quintanilla JAV, Zuccolotto LEM (2011). Ind. Crops Prod..

[5] Maswada HF, Abdallah S (2013). J. Biol. Sci..

[6] Silva FM, Paula JE, Espindola LS (2009). Mycoses.

[7] Santos BR, Paiva R, Dombroski JLD, Martinotto C, Nogueira RC, Silva AAN (2004). Bol. Agrop. Univ. Federal de Lavras..

[8] Silva MR, Lacerda DBCL, Santos GG, Martins DMO (2008). Ciênc. Rural..

[9] Agostini-Costa TS, da Silva DB, Vieira RF, Sano SM, Ferreira FR, Vieira RF, Agostini-Costa TS, da Silva DB, Sano SM, Ferreira FR (2010). Frutas nativas da região Centro-Oeste do Brasil.

[10] Passos XS, Santos SC, Ferri PH, Fernandes OFL, Paula TF, Garcia ACF, Silva MRR (2002). Rev. Soc. Bras. Med. Trop..

[11] Coutinho ID, Cardoso CAL, Ré-Poppi N, Melo AM, Vieira MC, Honda NK, Coelho RG (2009). Braz. J. Pharm. Sci..

[12] Dapkevicius A, Venskutonis R, Beek TAV, Linssen J (1998). J. Sci. Food Agric..

[13] Duarte MCT, Figueira GM, Sartoratto A, Rehder VLG, Delarmelina C (2005). J. Ethnopharmacol..

[14] Bashir HA, Abu-Goukh AA (2003). Food Chem..

[15] Donlan RM, Costerton JW (2002). Clin. Microbiol. Rev..

[16] Baert K, Valero A, de Meulenaer B, Samapundo S, Ahmed MM, Bo L, Debevere J, Devlieghere F (2007). Int. J. Food Microbiol..

[17] Nychas GJE, Gould GW (1995). New methods of food preservation.

[18] de la Luz Cádiz-Gurrea M, Fernández-Arroyo S, Joven J, Segura-Carretero A (2013). Food Res. Int..

[19] Huang ST, Wang CY, Yang RC, Wu HT, Yang SH, Cheng YC, Pang JHS (2011). Altern. Med..

[20] Lee JH, Johnson JV, Talcott ST (2005). J. Agric. Food Chem..

[21] Roesler R, Catharino RR, Malta LG, Eberlin MN, Pastore G (2008). Food Chem..

[22] Jayamani J, Shanmugam G (2014). Eur. J. Med. Chem..

[23] Bhouri W, Boubaker J, Skandrani I, Ghedira K, Ghedira LC (2012). Cancer Cell Int..

[24] Osorio E, Flores M, Hernández D, Ventura J, Rodríguez R, Aguilar CN (2010). Ind. Crops Prod..

[25] Barco A, Benetti S, Risi CD, Marchetti P, Pollini GP, Zanirato V (1997). Tetrahedron Asymmetry.

[26] Chen J, Yang ML, Zeng J, Gao K (2013). Phytochem. Lett..

[27] Tian J, Xie Y, Zhao Y, Li C, Zhao S (2011). Luminescence.

[28] N’Guessan JD, Bidié AP, Lenta BN, Weniger B, André P, Guédé-Guina F (2007). African. J. Biotechnol..

[29] Pinto MS, Lajolo FM, Genovese MI (2008). Food Chem..

[30] Buzzini P, Arapitsas P, Goretti M, Branda E, Turchetti B, Pinelli P, Ieri F, Romani A (2008). Mini Rev. Med. Chem..

[31] Latté KP, Kolodziej H (2000). Z. Naturforschung C..

[32] Wang Z, Guo QY, Zhang XJ, Li X, Li WT, Ma XT, Ma LJ (2014). Int. J. Mol. Sci..

[33] Perera A, Appleton D, Ying LH, Elendran S, Palanisamy UD (2012). Sep. Purif. Technol..

[34] Taguri T, Tanaka T, Kouno I (2004). Biol. Pharm. Bull..

[35] Organization for Economic Co-operation and Development, OECD guideline for testing of chemicals—guideline no 420 (2001)

[36] El-Arini SK, Leuenberger H (1998). Pharm. Acta Helv..

[37] National Commitee for Clinical Laboratory Standards, NCCLS document M38-A (2002)

[38] Gougoli M, Koutsoumanis KP (2013). Int. J. Food Microbiol..

